# Protocol for transcriptome assembly by the TransBorrow algorithm

**DOI:** 10.1093/biomethods/bpad028

**Published:** 2023-11-01

**Authors:** Dengyi Zhao, Juntao Liu, Ting Yu

**Affiliations:** School of Mathematics and Statistics, Shandong University, Weihai 264209, China; School of Mathematics and Statistics, Shandong University, Weihai 264209, China; Research Center for Mathematics and Interdisciplinary Sciences, Shandong University, Qingdao 266237, China

**Keywords:** RNA-seq data, transcriptome assembly, splice variants

## Abstract

High-throughput RNA-seq enables comprehensive analysis of the transcriptome for various purposes. However, this technology generally generates massive amounts of sequencing reads with a shorter read length. Consequently, fast, accurate, and flexible tools are needed for assembling raw RNA-seq data into full-length transcripts and quantifying their expression levels. In this protocol, we report TransBorrow, a novel transcriptome assembly software specifically designed for short RNA-seq reads. TransBorrow is employed in conjunction with a splice-aware alignment tool (e.g. Hisat2 and Star) and some other transcriptome assembly tools (e.g. StringTie, Cufflinks, and Scallop). The protocol encompasses all necessary steps, starting from downloading and processing raw sequencing data to assembling the full-length transcripts and quantifying their expressed abundances. The execution time of the protocol may vary depending on the sizes of processed datasets and computational platforms.

## Introduction

RNA-seq experiments involve several key steps. First, RNA extraction is performed in the laboratory to obtain mRNAs or noncoding RNAs, followed by cDNA reverse transcription, and preparation of sequencing libraries with ligated junctions [1]. Subsequently, these libraries are sequenced using high-throughput platforms such as Illumina, generating a large number of raw sequenced reads with a shorter read length [2]. Therefore, one of the most critical steps for RNA-seq data analysis is computationally assembling the tremendous amount of sequencing reads into full-length transcripts, which are generally categorized into two kinds of strategies: *de novo* and genome guided [3]. For the model species (e.g. human and mouse) where a high-quality reference genome is available, a genome-guided strategy is usually used, which involves aligning these sequenced reads to a reference genome with a splice-aware aligner, and then assembling full-length transcripts based on the aligning results [4]. Additionally, the transcripts are quantified via using computational models to assign the aligned reads to the predicted transcripts [5].

Assembling high-throughput sequencing reads into full-length transcripts poses a significant challenge due to the large quantity, short read length, and sequencing errors of the raw sequencing data. Besides, the complexity of the alternative splicing events during gene expressions also presents huge difficulties for transcriptome assembly [6]. Therefore, accurate and efficient assembly tools are needed to achieve the full potential of RNA-seq technology in this setting [7].

In this protocol, we report TransBorrow, a novel genome-guided transcriptome assembler. TransBorrow leverages the assembled results from other transcript assembly tools to guide the assembly procedure and implements an effective path extension method to identify transcript-representing paths on each splice graph [8]. Our results demonstrate that TransBorrow outperforms other assembly tools of its kind in terms of accuracy and efficiency.

**Figure 1.**
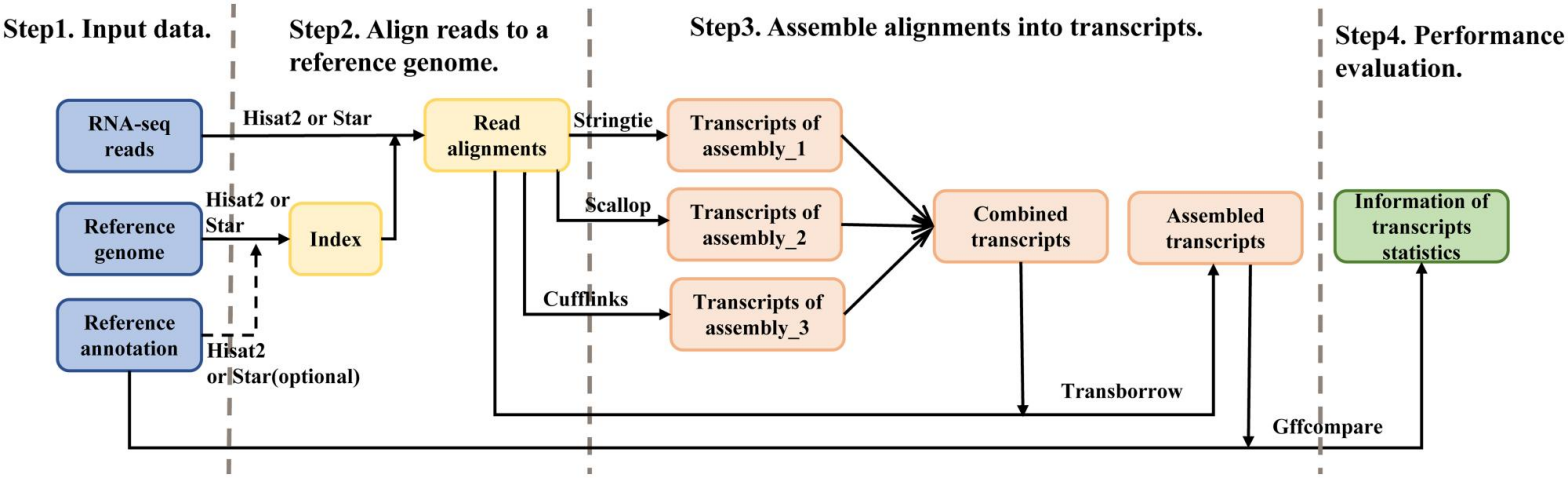
Workflow of transcriptome assembly of RNA-seq experiments with TransBorrow.

To validate the accuracy and efficiency of TransBorrow in assembling full-length transcripts, we employed the following steps [9]. We began by downloading the raw sequencing data and utilized the state-of-the-art mapping tool HISAT2 to map the RNA-seq reads to a reference genome [10]. Subsequently, based on the mapping results, we run several leading assemblers, including Scallop, StringTie, and Cufflinks, to assemble the transcripts in the test datasets [11–13], and then we run TransBorrow by taking as input the mapping results and the assemblies generated from the three aforementioned assemblers. Finally, we used the Gffcompare tool to determine the number of correctly assembled transcripts [9]. This protocol outlines the process of the download and installation of the necessary software on a Linux system, and the step-by-step usage of TransBorrow for assembling full-length transcripts in RNA-seq experiments [14] (see Fig. 1). This protocol is specifically designed for researchers who are not good at any programming skills but are familiar with the Linux command-line interface and able to run basic Linux simple commands.

## Materials and methods

### Equipment

#### Data

Raw RNA-seq data (https://www.ncbi.nlm.nih.gov/sra), the corresponding reference genome, and transcriptome (http://genome.ucsc.edu/cgi-bin/hgTables).

Sratoolkit (https://ftp-trace.ncbi.nlm.nih.gov/sra/sdk, version 3.0.6 or later).

Hisat2 (http://github.com/infphilo/hisat2, version 2.2.1 or later).

Star (https://github.com/alexdobin/STAR, version 2.5.3 or later).

Samtools (https://nchc.dl.sourceforge.net/project/samtools/samtools, version 1.1.7 or later).

StringTie (http://ccb.jhu.edu/software/stringtie, version 2.2.1 or later).

Cufflinks (http://cole-trapnell-lab.github.io/cufflinks, version 2.2.1 or later).

Scallop (https://github.com/Kingsford-Group/scallop, version 0.10.5 or later).

Boost (https://boostorg.jfrog.io/artifactory/main/release/1.82.0/source, version 1.82.0 or later).

Transborrow (https://sourceforge.net/projects/transcriptomeassembly/files/TransBorrow, version 1.3 or later).

Gffcompare (https://github.com/gpertea/gffcompare, version 0.12.6 or later).

TACO (https://github.com/tacorna/taco, version 0.7.3 or later).

Hardware (64-bit computer running Linux; 4 GB of RAM, preferably 8 GB or higher).

### Software downloading and installation

It is crucial to note that these instructions assume a Linux environment and the availability of the chosen binary packages. If an alternative compressed package or different operating system is used, additional steps may be required to configure the software accordingly. Thus, to simplify the setup process, it is suggested to select a binary package of the software under Linux environments.

#### Sratoolkit software downloading and installation

The Sratoolkit is a comprehensive toolset offered by the National Center for Biotechnology Information (NCBI) to process and analyze high-throughput sequencing data. This toolkit encompasses a range of command-line utilities specifically crafted to aid researchers in accessing, downloading, and converting the format of raw sequencing data stored within the NCBI Sequence Read Archive (SRA) database [15].

To install the Sratoolkit, the following steps can be followed. First, create a subdirectory named “Sratoolkit” within your specified directory. Subsequently, navigate to the “Sratoolkit” directory and employ the “wget” command to download the latest package from the appropriate downloading link address. Then, extract the contents of the downloaded package to obtain the executable files. Lastly, add the path of the Sratoolkit binaries to your system’s PATH variable in the shell configuration file, which is generally ∼/.bashrc if you are using a bash shell (see the following illustration). Alternatively, you may directly download the installation package from the official website and move it to the current directory. The prefetch tool can be utilized to download raw sequencing data (in .sra format), and the fastq-dump tool can be used to convert SRA files into FASTQ format.

An illustration of installing Sratoolkit is shown as follows.

$ mkdir Sratoolkit

$ cd Sratoolkit

$ wget https://ftp-trace.ncbi.nlm.nih.gov/sra/sdk/3.0.6/sratoolkit.3.0.6-ubuntu64.tar.gz

$ tar -xzvf sratoolkit.3.0.6-ubuntu64.tar.gz

$ echo “export PATH=$PATH:/mnt/data0/zhaody/Sratoolkit/sratoolkit.3.0.6-ubuntu64/bin”≫∼/.bashrc

$ source ∼/.bashrc

After you successfully install it, you can type the command “prefetch -h” to see the help documentation information.

#### Hisat2 software downloading and installation

Hisat2 is a powerful splice-aware aligner specifically designed for aligning short RNA-seq reads to a reference genome, which uses an indexing scheme based on the Burrows–Wheeler transform and the Ferragina–Manzini (FM) index, employing two types of indexes for alignment: a whole-genome FM index to anchor each alignment and numerous local FM indexes for very rapid extensions of these alignments. The alignment file generated by Hisat2 is in sequence alignment map (SAM) format, which can be subsequently converted into binary alignment map (BAM) format, and taken as the input for the assembly tools [10, 16].

To install Hisat2, the subsequent steps can be followed. First, create a subdirectory “Hisat2” in your directory and enter it. Next, employ the “wget” command to download the most recent package from the appropriate downloading link address. Subsequently, extract the contents of the downloaded package to obtain the executable file. Lastly, add the path of the Hisat2 binary to a directory encompassed within your system’s PATH variable.

An illustration of installing Hisat2 is shown as follows.

$ mkdir Hisat2

$ cd Hisat2

$ wget https://cloud.biohpc.swmed.edu/index.php/s/oTtGWbWjaxsQ2Ho/download

$ unzip download

$ echo “export PATH=$PATH:/mnt/data0/zhaody/Hisat/hisat2-2.2.1”≫∼/.bashrc

$ source ∼/.bashrc

If you have successfully installed it, you can type the command “hisat2” to see the help documentation information.

#### Star software downloading and installation

Star is a tool for aligning sequencing data (especially RNA-seq data) to a reference genome. It efficiently handles splice variants and insertion/deletion events in the genome based on a previously un-described RNA-seq alignment algorithm which utilizes sequential maximum mappable seed search in uncompressed suffix arrays followed by seed clustering and stitching procedure [17].

To install Star, the subsequent steps can be followed. First, create a subdirectory “Star” in your directory and enter it. Next, employ the “wget” command to download the most recent package from the appropriate downloading link address. Subsequently, extract the contents of the downloaded package to obtain the executable file. Lastly, add the path of the Star binary to a directory encompassed within your system’s PATH variable.

An illustration of installing Star is shown as follows.

$ mkdir Star

$ cd Star

$ wget https://github.com/alexdobin/STAR/archive/2.5.3a.tar.gz

$ tar -xzf 2.5.3a.tar.gz

$ cd STAR-2.5.3a

$ echo “export PATH=$PATH:/mnt/data0/zhaody/Star/STAR-2.5.3a”≫∼/.bashrc

$ source ∼/.bashrc

#### Samtools software downloading and installation

Samtools is a software package designed for processing SAM and BAM format files, which offers a diverse range of command-line utilities for manipulating, converting, and analyzing SAM/BAM files. It enables various tasks such as file format conversion (e.g. SAM to BAM conversion), sorting and indexing SAM/BAM files, assessing coverage and depth of aligned loci, extracting sequences from specific regions, and more [18].

To install Samtools, the following steps can be undertaken. First, create a subdirectory named “Samtools” within your directory. Subsequently, navigate to the “Samtools” directory and use the “wget” command to download the latest package from the appropriate download link address. Afterwards, extract the downloaded package and configure the environment variables for Samtools accordingly. Finally, add the Samtools binary directory to the PATH environment variable in the shell configuration file (see the following illustration)

An illustration of installing samtools is shown as follows.

$ mkdir Samtools

$ cd Samtools

$ wget https://nchc.dl.sourceforge.net/project/samtools/samtools/1.17/samtools-1.17.tar.bz2

$ tar jxvf samtools-1.17.tar.bz2

$ cd samtools-1.17

$ ./configure—prefix=/mnt/data0/zhaody/Samtools/samtools-1.17

$ make

$ make install

$ echo “export PATH=$PATH:/mnt/data0/zhaody/Samtools/samtools-1.17”≫∼/.bashrc

$ source ∼/.bashrc

After installing it, you can type the command “samtools” to see the help documentation information.

#### StringTie software downloading and installation

StringTie is an efficient transcriptome assembler, which iteratively extracts the heaviest path from a splice graph, and then estimates the abundance via a network flow algorithm [12].

To install StringTie, the following steps can be followed. First, create a subdirectory named “StringTie” within your directory. Next, navigate to the “StringTie” directory and utilize the “wget” command to download the latest package from the appropriate download link address. After downloading, extract the contents of the package to obtain the executable file. Finally, add the path of the StringTie binary directory to the PATH environment variable in the shell configuration file.

An illustration of installing StringTie is shown as follows.

$ mkdir StringTie

$ cd StringTie

$ wget http://ccb.jhu.edu/software/stringtie/dl/stringtie-2.2.1.Linux_x86_64.tar.gz

$ tar -xzvf stringtie-2.2.1.Linux_x86_64.tar.gz

$ echo “export PATH=$PATH:/mnt/data0/zhaody/Stringtie/stringtie-2.2.1.Linux_x86_64”≫∼/.bashrc

$ source ∼/.bashrc

If you have successfully installed it, you can type the command “stringtie -h” to see the help documentation information.

#### Cufflinks software downloading and installation

Cufflinks is a software tool specifically designed for analyzing RNA-seq data. It constructs the overlap graph model based on the fragment alignments, and applies a minimum path cover model to search for the transcript-representing paths [13].

To install Cufflinks, the following steps can be undertaken: First, create a subdirectory named “Cufflinks” within your directory. Next, navigate to the “Cufflinks” directory and use the command “wget” to download the latest package from the appropriate download link address. After downloading the package, extract its contents to obtain the executable file. Finally, add the Cufflinks binary directory to the PATH environment variable in the shell configuration file.

An illustration of installing Cufflinks is shown as follows.

$ mkdir Cufflinks

$ cd Cufflinks

$ wget http://cole-trapnell-lab.github.io/cufflinks/assets/downloads/cufflinks-2.2.1.Linux_x86_64.tar.gz

$ tar -xzvf cufflinks-2.2.1.Linux_x86_64.tar.gz

$ echo “export PATH=$PATH:/mnt/data0/zhaody/Cufflinks/cufflinks-2.2.1.Linux_x86_64”≫∼/.bashrc

$ source ∼/.bashrc

If you have successfully installed it, you can type the command “cufflinks -h” to see the help documentation information.

#### Scallop software downloading and installation

Scallop is a highly efficient transcriptome assembly software designed for the reconstruction of transcripts from RNA-Seq data. It was built upon the standard paradigm of the splice graph, and it decomposed the graphs through optimizing several competing objectives while preserving long-range phasing paths [11].

To install Scallop, the following steps can be followed. First, create a subdirectory named “Scallop” within the main directory. Next, navigate to the “Scallop” directory and utilize the command “wget” to download the latest package from the appropriate download link address. After downloading, extract the contents of the package to obtain the executable file. Finally, add the Scallop binary directory to the PATH environment variable in the shell configuration file.

An illustration of installing Scallop is shown as follows.

$ mkdir Scallop

$ cd Scallop

$ wget https://github.com/Kingsford-Group/scallop/releases/download/v0.10.5/scallop-0.10.5_linux_x86_64

.tar.gz

$ tar -xzvf scallop-0.10.5_linux_x86_64.tar.gz

$ echo “export PATH=$PATH:/mnt/data0/zhaody/Scallop/scallop-0.10.5_linux_x86_64”≫∼/.bashrc

$ source ∼/.bashrc

If you have successfully installed it, you can type the command “scallop -h” to see the help documentation information.

#### BOOST downloading and installation

The Boost library is a highly regarded, portable, and open-source C++ library that is essential for TransBorrow. To install Boost, follow these steps. First, create a subdirectory named “Boost” under your directory. Next, navigate into the “Boost” directory and use the command “wget” to download the latest package from the appropriate download link address. Then, unzip the downloaded package and change to the *boost* directory and run “./bootstrap.sh”. Finally, type “./b2 install—prefix=<YOUR_BOOST_INSTALL_DIRECTORY>” to install Boost (please replace <YOUR_BOOST_INSTALL_DIRECTORY> with the desired installation directory for Boost).

Please note that the version number and URL provided in your original text may need to be updated. Make sure to use the latest version of Boost and adjust the URL accordingly.

An illustration of installing Boost is shown as follows.

$ mkdir Boost

$ cd Boost

$ wget https://boostorg.jfrog.io/artifactory/main/release/1.82.0/source/boost_1_82_0.tar.gz

$ tar -xzvf boost_1_82_0.tar.gz

$ cd boost_1_82_0

$ ./bootstrap.sh

$ ./b2 install—prefix=/mnt/data0/zhaody/Boost

If the Boost is installed successfully, you can see the “lib” and “include” directories in YOUR_BOOST_INSTALL_DIRECTORY directory. Take note of the Boost installation directory, because you need to tell the TransBorrow installer where to find Boost later on.

#### TransBorrow software downloading and installation

TransBorrow is an accurate and efficient transcriptome algorithm, which borrows the assemblies from different assemblers to search for reliable subsequences by building a colored graph from those borrowed assemblies, and employs a newly designed path extension strategy to accurately search for a transcript-representing path cover over each splicing graph [8].

To install TransBorrow, create a subdirectory named TransBorrow under the main directory. After entering the directory, use the command “wget” to download a package from the appropriate download link address and unzip it. In addition, to configure the environment of TransBorrow, change to the “bamtools” directory and make a new directory named “build”, then type “cmake” and “make” to make it install.

An illustration of building bamtools is shown as follows.

$ mkdir TransBorrow

$ cd TransBorrow

$ wget https://sourceforge.net/projects/transcriptomeassembly/files/TransBorrow/TransBorrow_v.1.3.tar.gz

$ tar -xzvf TransBorrow_v.1.3.tar.gz

$ cd TransBorrow_v.1.3

$ cd bamtools

$ mkdir build

$ cd build

$ cmake ./

$ make

$ cd ./

Assuming the build process finished correctly, you should be able to find the toolkit executable in directory “./bin/”, the Bamtools API Utils libraries “./lib/”, and the Bamtools API headers “./include/”.

Then, add the *lib* and *include* directories (absolute path) of both Boost and bamtools to the CMakeLists.txt file located in TransBorrow_v.1.3/src/to configure the installation environment for TransBorrow (see the following illustration for details).

Please note that the directory structure and file names may vary depending on your specific setup. Adjust the paths and commands accordingly.

An illustration of setting the installation environment for TransBorrow is shown as follows.

$ cd src

$ vim CMakeList.txt

**set(BOOST_LIB_DIR/mnt/data0/zhaody/Boost/lib)**

#set(BOOST_LIB_DIR/storage/juntaosdu/razor-home/juntaosdu/local/boost/lib)

**set(BOOST_INCLUDE_DIR/mnt/data0/zhaody/Boost/include)**

#set(BOOST_INCLUDE_DIR/storage/juntaosdu/razor-home/juntaosdu/local/boost/include)

**set(BAMTOOLS_LIB_DIR/mnt/data0/zhaody/TransBorrow/TransBorrow_v.1.3/bamtools/lib)**

#set(BAMTOOLS_LIB_DIR/storage/juntaosdu/yuting/bamtools/lib)

**set(BAMTOOLS_INCLUDE_DIR/mnt/data0/zhaody/TransBorrow/TransBorrow_v.1.3/bamtools/include)**

#set(BAMTOOLS_INCLUDE_DIR/storage/juntaosdu/yuting/bamtools/include)

Change to the TransBorrow root directory and make a new directory named “build” and change into it, then type “cmake ./src” and “make” commands for the final installation of TransBorrow.

An illustration of building TransBorrow is shown as follows.

$ cd ../

$ mkdir build

$ cd build

$ cmake ./src

$ make

$ cd ./src

$ make

Finally, add the TransBorrow directory to the PATH environment variable in the shell configuration file. See the following illustration.

$ cd TransBorrow

$ echo “export PATH=$PATH:/mnt/data0/zhaody/TransBorrow/TransBorrow_v.1.3/build”≫∼/.bashrc

$ source ∼/.bashrc

**Figure 2.**
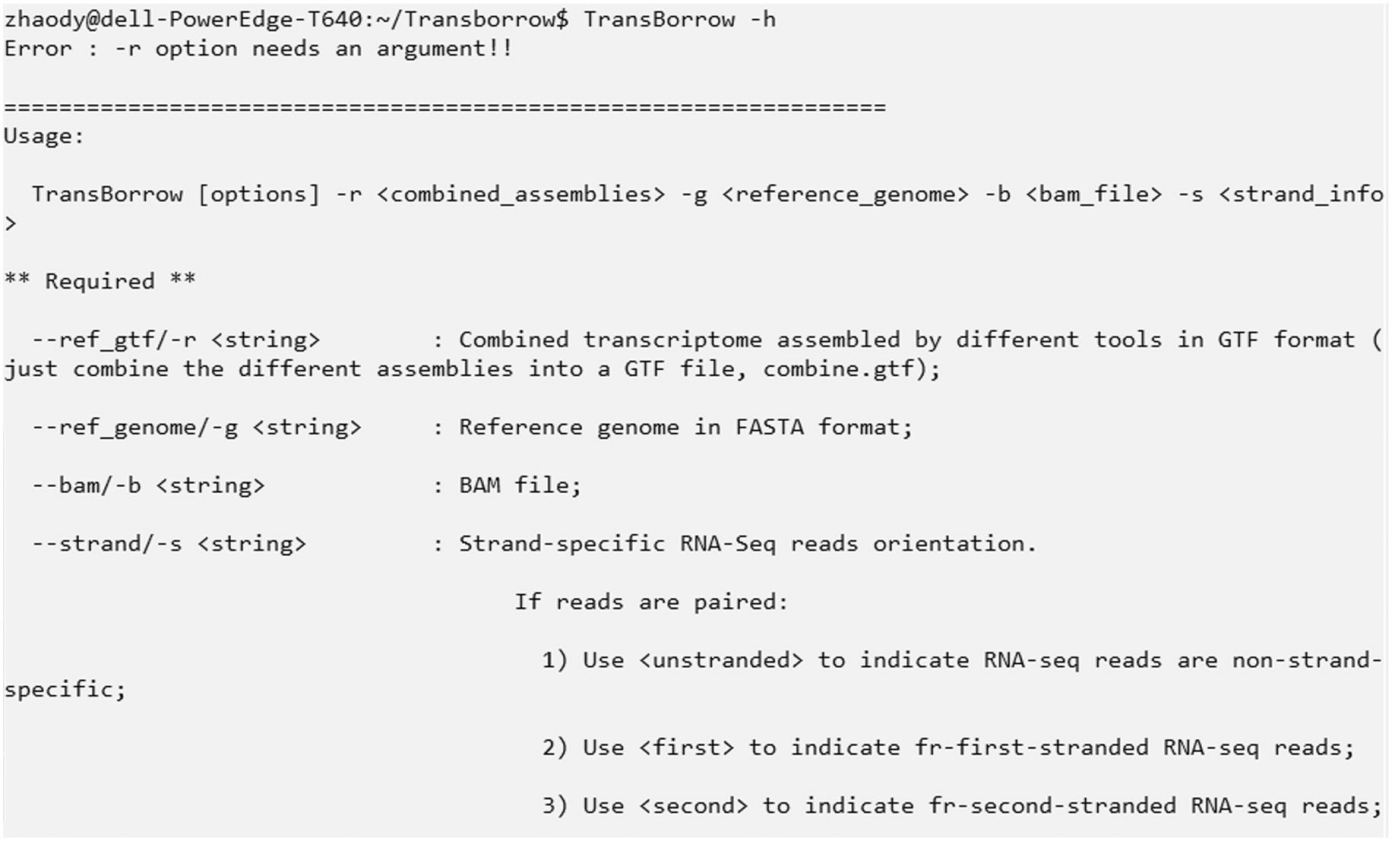
The help information of TransBorrow.

If you have successfully installed it, you can type the command “TransBorrow -h” to see the help documentation information (see Fig. 2).

#### Gffcompare software downloading and installation

The Gffcompare software is a powerful tool utilized for comparing, merging, annotating, and estimating the accuracy of one or more GFF/GTF files in comparison to reference annotations [9]. To install Gffcompare, the following steps should be followed. First, create a subdirectory named “Gffcompare” within the main directory. Subsequently, navigate into the “Gffcompare” folder and utilize the “wget” command to download the latest package from the appropriate download link address. Next, extract the downloaded package to acquire the executable file. Finally, add the Gffcompare binary directory to the PATH environment variable.

An illustration of installing Gffcompare is shown as follows.

$ mkdir Gffcompare

$ cd Gffcompare

$ wget http://ccb.jhu.edu/software/stringtie/dl/gffcompare-0.12.6.Linux_x86_64.tar.gz

$ tar -xzvf gffcompare-0.12.6.Linux_x86_64.tar.gz

$ echo “export PATH=$PATH:/mnt/data0/zhaody/Gffcompare/gffcompare-0.12.6.Linux_x86_6”≫∼/.bashrc

$ source ∼/.bashrc

If you have successfully installed it, you can type the command “gffcompare -h” to see the help documentation information.

#### TACO software downloading and installation

TACO is a tool to reconstruct a consensus transcriptome from multiple RNA-seq data sets. TACO accepts as input a set of GTF files containing transcripts assembled from individual libraries, and it employs a dynamic programming path search strategy in the path graph to reconstruct the transcripts. To install TACO, the following steps should be followed. First, create a subdirectory named “TACO” within the main directory. Subsequently, navigate into the “TACO” folder and utilize the “wget” command to download the latest package from the appropriate download link address. Next, extract the downloaded package to acquire the executable file. Finally, add the TACO binary directory to the PATH environment variable.

An illustration of installing TACO is shown as follows.

$ mkdir TACO

$ cd TACO

$ wget wget https://github.com/tacorna/taco/releases/download/v0.7.3/taco-v0.7.3.Linux_x86_64.tar.gz

$ tar -xzvf taco-v0.7.3.Linux_x86_64.tar.gz

$ echo “export PATH=$PATH:/mnt/data0/zhaody/TACO/taco-v0.7.3.Linux_x86_64”≫∼/.bashrc

$ source ∼/.bashrc

### Obtaining data

The RNA-seq data can be downloaded from databases such as the NCBI or the European Bioinformatics Institute (EBI), etc. Alternatively, the researchers can use the RNA-seq data sequenced by themselves. Note that the file format of the input sequencing data must be in FASTA or FASTQ format.

RNA-seq technology is mainly categorized into single-end sequencing and paired-end sequencing, based on differences in DNA library preparation during the sequencing process. Single-end sequencing means sequencing only one end of the target DNA/RNA, which requires less sequencing time and cost. In comparison, paired-end sequencing refers to sequencing both ends of the target DNA/RNA, which improves the accuracy of mapping and assembly [1]. Additionally, RNA-seq technology can be classified into strand-specific sequencing and nonstranded sequencing based on differences in cDNA strand synthesis during the sequencing process. Strand-specific sequencing differentiates the forward and reverse strand information of the target transcript during RNA sequencing, thereby enabling more accurate quantification and localization of gene transcription products. Conversely, nonstranded sequencing involves the absence of forward and reverse strand information in all reads obtained during RNA-seq, making it a less costly method for RNA-seq [2].

Therefore, the proposed protocol utilized three human RNA-seq data sets based on different types of RNA-seq technology (see Table 1) to demonstrate the complete running process of the TransBorrow software. All three datasets were downloaded from the NCBI SRA database using the Sratoolkit with the following steps. First, utilize the “prefetch” command to download the raw data. Next, use the “fastq-dump” command to decompress the files for each sample (see the following illustration for details).

An illustration of downloading sequencing data is shown as follows.

**Table 1.** Summary of the RNA-seq data sets used in this protocol

SRA accession code	# of Spots	Read length	Species	Sequencing protocol	Layout
SRR7807492	36,080,337	100	Homo sapiens	Nonstranded	Paired-end
ERR3639851	29,167,280	131	Homo sapiens	Nonstranded	Single-end
SRR10611964	23,892,110	^a^	Homo sapiens	Strand-specific	Paired-end

a The dataset contains reads with different lengths.

$ cd Sratoolkit

$ prefetch SRR7807492

$ cd SRR7807492

$ fastq-dump—split-3 SRR7807492.sra

$ prefetch ERR3639851

$ cd ERR3639851

$ fastq-dump—split-3 ERR3639851.sra

$ prefetch SRR10611964

$ cd SRR10611964

$ fastq-dump—split-3 SRR10611964.sra

After obtaining raw RNA-seq reads, download the human reference genome (the version used in this protocol was hg19) as follows. Create a file named “ref_genome” in your home directory and utilize the “wget” command with the appropriate download link address to download the corresponding file. If you complete the downloading, extract the file (see the following illustration for details).

$ mkdir ref_genome

$ cd ref_genome

$ wget http://hgdownload.soe.ucsc.edu/goldenPath/hg19/bigZips/chromFa.tar.gz

$ gunzip *.gz

$ cat *.fa > ref_genome.fa

$ rm chr*.fa

The human genome is about 3GB and ensures that you have sufficient storage capacity to accommodate both the downloaded and uncompressed files.

In this protocol, all the reference transcripts were set as the ground truth to evaluate the performance of the assemblers. To acquire the reference transcripts, navigate to the “ref_genome” directory and employ the “wget” command along with the appropriate downloading link address (see the following illustration for details).

An illustration of downloading the reference transcriptome is shown below.

$ cd ref_genome

$ wget https://ftp.ebi.ac.uk/pub/databases/gencode/Gencode_human/release_44/GRCh37_mapping/gencode.v44lift37.annotation.gtf.gz

$ gunzip gencode.v44lift37.annotation.gtf.gz

**Figure 3.**
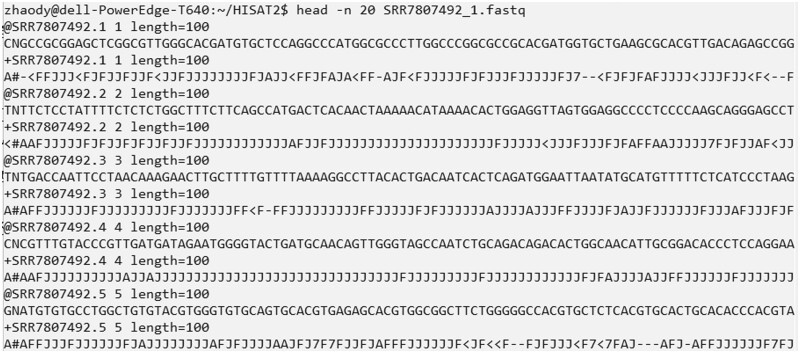
A snapshot of 20 lines in the SRR7807492_1.fastq file that stores the sequencing reads. Each four lines contains the information about one sequencing read. The first line begins with a “@” character followed by a sequence identifier and an optional description. The second line contains the raw sequence letters. The third line begins with a “+” character optionally followed by the same sequence identifier. The fourth line records the sequencing quality values for the sequence.

Upon successful downloading, you will find three folders named SRR7807492, ERR3639851, and SRR10611964 within the Sratoolkit directory, which store the RNA-seq data in the “.fastq” format (see Fig. 3 for an example of the data). Additionally, within the ref_genome directory, you will find the reference genome in “.fa” format and the annotated transcriptome in “.gtf” format. These files will be utilized for the subsequent transcript assembly and performance evaluations.

### Mapping reads to a reference genome with Hisat2 or Star

To align reads to a reference genome, effective splice-aware mapping tools such as Hisat2 or Star can be used. Hisat2 is well-known for its ability to produce precise and efficient results while utilizing less memory during the mapping process. STAR can handle splicing variants with a high degree of accuracy and flexibility, and is capable of handling large-scale RNA-seq data quickly and efficiently. In this protocol, we provide a detailed description of how to utilize Hisat2 and STAR to align reads to a reference genome and generate read alignments, respectively.

#### Using Hisat2 to complete the mapping task

The following are some parameter descriptions and suggestions for using Hisat2 to complete the mapping tasks.

1) The “hisat2-build” command generates the reference genome index and is typically followed by the reference genome file in “.fa” format and the desired output file name. The recommended command for this task is as follows.

# hisat2-build ref_genome.fa ref_index_genome,

where *ref_genome.fa* is the reference genome.

Besides, when utilizing a transcriptome annotation library to aid in building the reference genome index, “hisat2-build” requires the use of the “—ss” parameter, which specifies the splice site information of the reference transcripts, and the “—exon” parameter, which denotes the exon information of the reference transcripts. The running command should be as follows.

# hisat2-build—ss genome.ss—exon genome.exon ref_genome.fa ref_index_genome,

where *genome.ss* and *genome.exon* can be extracted from a transcriptome annotation file (in GTF format) using the script “extract_splice_sites.py” and “extract_exons.py” provided in the Hisat2 package.

2) The command “hisat2” is utilized for aligning RNA-seq reads to the reference genome. The recommended command is as follows.

# hisat2 -p 8—dta -x ref_index_genome -1 reads_1.fastq -2 reads_2.fastq -S test_genome.sam where the parameter “-p” specifies the number of threads running, typically set to be equal to or slightly fewer than the number of available CPU cores. The parameter “—dta” means reporting alignments tailored for transcript assemblers. The parameters “-x” and “-S” provide the indexed reference genome and the name of the output SAM file, respectively. The parameters “-1” and “-2” specify the two paired-end sequencing files in FASTQ format, reads_1.fastq and reads_2.fastq, respectively. If the sequencing data is single-ended, the parameter “-U” is used with the corresponding FASTQ file.

##### Construction of an index to the reference genome with Hisat2

The first step of using Hisat2 is to construct an index for the reference genome. Begin by navigating to the Hisat2 directory and employing the “mv” command to relocate the reference genome file from the “ref_genome” directory to the current directory. Subsequently, utilize the “hisat2-build” command to generate an index for the reference genome. This process will result in the creation of eight files with the “.ht2” suffix. This process of building the index typically takes approximately half an hour (see the following illustration for details).

An illustration of the Hisat2 building index is shown below.

$ cd Hisat2

$ mv/mnt/data0/zhaody/ref_genome/ref_genome.fa.

$ hisat2-build ref_genome.fa ref_index_genome

It is noteworthy that incorporating the annotation information of the transcripts while building the genome index can improve the accuracy of Hisat2 alignment. Utilizing this approach is also an option as follows. Navigate to the Hisat2 directory and employ the “mv” command to relocate the reference genome file and the transcript annotation file from the “ref_genome” directory to the current directory. Then, extract splice-site and exon information from the annotation. Proceed by using the “hisat2-build” command to construct an index for the reference genome. Finally, eight files will be generated with the “.ht2” suffix. This process of building the index typically takes approximately one hour (see the following illustration for details).

An illustration of the Hisat2 building index using the annotation file is shown below.

$ cd Hisat2

$ mv/mnt/data0/zhaody/ref_genome/ref_genome.fa.

$ mv/mnt/data0/zhaody/ref_genome/gencode.v44lift37.annotation.gtf.

$ extract_splice_sites.py gencode.v44lift37.annotation.gtf >genome.ss

$ extract_exons.py gencode.v44lift37.annotation.gtf >genome.exon

$ hisat2-build—ss genome.ss—exon genome.exon ref_genome.fa ref_index_genome

##### Aligning RNA-seq reads to the reference genome with Hisat2

**Figure 4.**
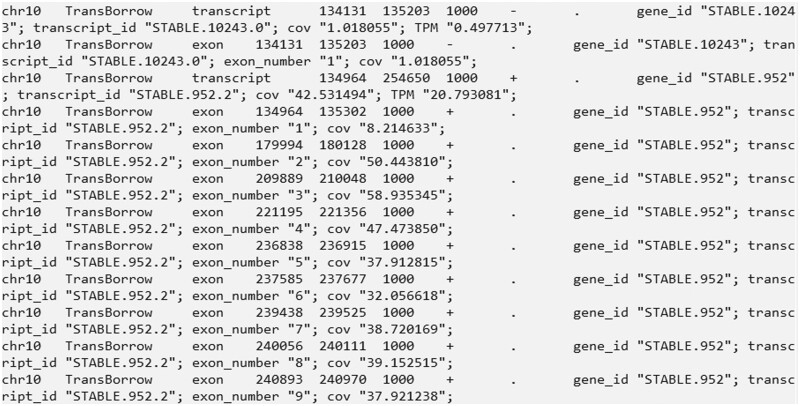
A snapshot of the first several lines in the SRR7807492_genome.sam file that stored the alignment information of RNA-seq reads to the reference genome. Lines starting with “@” are the headers of the file. Each line after the headers records the alignment information of one reads (e.g. query name, flag, reference sequence name, start position, CIGAR string, mate reference sequence name, mate start position, insert size, base sequence of the read, etc.).

To align the raw RNA-seq reads to the reference genome, first, navigate to the Hisat2 directory. Then, utilize the “mv” command to relocate the individual “.fastq” files of the three sample datasets from the “Sratoolkit” directory to the current directory. Afterward, use the “hisat2” command to align the RNA-seq reads from the three sample datasets to the reference genome. Upon completion of the alignment process, you will obtain three mapping files in “.sam” format, with each file corresponding to one of the sample datasets (see the following illustration for details and see Fig. 4 for an illustration of SAM file).

An illustration of Hisat2 mapping is shown below.

$ cd Hisat2.

$ mv/mnt/data0/zhaody/Sratoolkit/SRR7807492/SRR7807492_1.fastq.

$ mv/mnt/data0/zhaody/Sratoolkit/SRR7807492/SRR7807492_2.fastq.

$ mv/mnt/data0/zhaody/Sratoolkit/ERR3639851/ERR3639851.fastq.

$ mv/mnt/data0/zhaody/Sratoolkit/SRR10611964/SRR10611964_1.fastq.

$ mv/mnt/data0/zhaody/Sratoolkit/SRR10611964/SRR10611964_2.fastq.

$ hisat2 -p 8—dta -x ref_index_genome -1 SRR7807492_1.fastq -2 SRR7807492_2.fastq -S SRR7807492_genome.sam

$ hisat2 -p 8—dta -x ref_index_genome -U ERR3639851.fastq -S ERR3639851_genome.sam

$ hisat2 -p 8—dta -x ref_index_genome -1 SRR10611964_1.fastq -2 SRR10611964_2.fastq -S SRR10611964_genome.sam

##### Sorting and converting the SAM files to BAM files with Samtools

Hisat2 generated the alignment in SAM format, while transcriptome assemblers generally take as input the alignment files in sorted BAM format, the binary version of SAM files, which can be converted by the Samtools software as follows.

To accomplish the conversion, navigate to the Samtools directory and utilize the “mv” command to move the three mapping files stored in the Hisat2 directory to the current directory. Subsequently, employ the “samtools sort” command to perform the sorting and conversion of the sam files to bam files.

An illustration of samtool sort is shown below.

$ cd Samtools

$ mv/mnt/data0/zhaody/Hisat2/SRR7807492_genome.sam.

$ mv/mnt/data0/zhaody/Hisat2/ERR3639851_genome.sam.

$ mv/mnt/data0/zhaody/Hisat2/SRR10611964_genome.sam.

$ samtools sort -@ 8 -o SRR7807492_genome.bam SRR7807492_genome.sam

$ samtools sort -@ 8 -o ERR3639851_genome.bam ERR3639851_genome.sam

$ samtools sort -@ 8 -o SRR10611964_genome.bam SRR10611964_genome.sam

#### Using Star to complete the mapping task

The following are some parameter descriptions and suggestions for using Star to complete the mapping tasks.

1) The command “STAR—runMode genomeGenerate” is utilized for constructing the reference genome index. The recommended command is as follows.

# STAR—runThreadN 8—runMode genomeGenerate—genomeDir ./index—genomeFastaFiles ref_genome.fa—sjdbGTFfile reference_Transcripts.gtf—sjdbOverhang readlenth-1where the parameter “—runThreadN” denotes the number of running threads, typically set to be equal to or slightly fewer than the number of CPU cores available. The parameter “—genomeDir” specifies the output directory where the index file is stored. The parameters “—genomeFastaFiles” and “—sjdbGTFfile” indicate the reference genome and reference transcripts respectively. It is generally recommended to set the parameter “—sjdbOverhang” to be the length of sequencing reads minus one.

2) The command “STAR” is employed for aligning RNA-seq reads to the reference genome. The recommended command is as follows:

# STAR—runThreadN 8—genomeDir ./index—readFilesIn reads_1.fastq reads_2.fastq—outSAMtype BAM SortedByCoordinate

where the parameter “—runThreadN” indicates the number of threads running, typically set to be equal to or slightly fewer than the number of available CPU cores. The parameter “—genomeDir” specifies the output directory where the index file is stored. The parameter “—readFilesIn” provides the sequencing reads in FASTQ format, namely reads_1.fastq and reads_2.fastq. The parameter “—outSAMtype BAM SortedByCoordinate” is used to generate a sorted BAM file as the final mapping output, eliminating the need for using Samtools to convert the SAM file to BAM. By default, the final mapping file is saved in the current folder.

##### Construction of an index to a reference genome with Star

The first step of using Star is to construct an index for the reference genome. Beginning by entering the Star directory and employing the “mv” command to relocate the reference genome file and the transcript annotation file from the “ref_genome” directory to the current directory. Subsequently, utilize the “STAR” command to generate an index for the reference genome. (see the following illustration for details).

An illustration of the Star building index is shown below.

$ cd Star

$ mv/mnt/data0/zhaody/ref_genome/ref_genome.fa.

$ mv/mnt/data0/zhaody/ref_genome/gencode.v44lift37.annotation.gtf.

$ STAR—runThreadN 8—runMode genomeGenerate—genomeDir ./index—genomeFastaFiles ref_genome.fa—sjdbGTFfile gencode.v44lift37.annotation.gtf—sjdbOverhang 99

##### Aligning RNA-seq reads to the reference genome with Star

To align the raw RNA-seq reads to the reference genome with Star, please navigate to the STAR directory. Then, utilize the “mv” command to relocate the individual “.fastq” files of the three sample datasets from the “Sratoolkit” directory to the current directory. Afterward, use the “STAR” command to align the RNA-seq reads from the three sample datasets to the reference genome. Upon completion of the alignment process, you will obtain three mapping files in “BAM” format, with each file corresponding to one of the sample datasets. By default, the final mapping file is saved in the current folder.

An illustration of Star mapping is shown below.

$ cd STAR

$ mv/mnt/data0/zhaody/Sratoolkit/SRR7807492/SRR7807492_1.fastq.

$ mv/mnt/data0/zhaody/Sratoolkit/SRR7807492/SRR7807492_2.fastq.

$ mv/mnt/data0/zhaody/Sratoolkit/ERR3639851/ERR3639851.fastq.

$ mv/mnt/data0/zhaody/Sratoolkit/SRR10611964/SRR10611964_1.fastq.

$ mv/mnt/data0/zhaody/Sratoolkit/SRR10611964/SRR10611964_2.fastq.

$ STAR—runThreadN 8—genomeDir ./index—readFilesIn SRR7807492_1.fastq SRR7807492_2.fastq—outSAMtype BAM SortedByCoordinate

$ STAR—runThreadN 8—genomeDir ./index—readFilesIn ERR3639851.fastq—outSAMtype BAM SortedByCoordinate

$ STAR—runThreadN 8—genomeDir ./index—readFilesIn SRR10611964_1.fastq SRR10611964_2.fastq—outSAMtype BAM SortedByCoordinate

### Transcriptome assembly with TransBorrow

#### The parameters of TransBorrow

TransBorrow performs transcript assembly by utilizing the alignment of RNA-seq reads to the reference genome, as well as the assemblies generated from other assemblers (e.g. StringTie, Scallop, and Cufflinks). In this protocol, we utilize the read alignments generated by Hisat2 to illustrate the entire TransBorrow’s transcript assembly process.

It is worth noting that during the TransBorrow assembly process, specific parameters and commands need to be selected according to the data types and your needs to achieve optimal assembly results. Table 2 provides a detailed description of the parameters of TransBorrow. A typical command for TransBorrow command should be:

# TransBorrow [options] -r <combined_assemblies> -g <reference_genome> -b <bam_file> -s <strandness>

**Table 2.** The description of TransBorrow’s parameters.

Parameters	Description
—ref_gtf/-r <string>	Combined transcriptome assembled by different tools in GTF format (just combine the different assemblies into a GTF file, combine.gtf)
—ref_genome/-g <string>	Reference genome in FASTA format;
—bam/-b <string>	BAM file;
—strand/-s <string>	Strand-specific RNA-Seq reads orientation. If reads are paired: 1) Use <unstranded> to indicate RNA-seq reads are nonstrand-specific; 2) Use <first> to indicate fr-first-stranded RNA-seq read; 3) Use <second> to indicate fr-second-stranded RNA-seq reads; If reads are single: 1) Use <single_unstranded> to indicate RNA-seq reads are nonstrand-specific; 2) Use <single_forward> to indicate RNA-seq reads forward; 3) Use <single_reverse> to indicate RNA-seq reads reverse;
—min_cov/-c <float>	Min coverage of recovered transcripts, default: 1;
—output/-o <string>	Output path, default: ./TransBorrow_results/TransBorrow.gtf;
—min_trans_len/-l <int>	Min length of recovered transcripts, default: 200;
—cre_num/-n <int>	Credible sub_paths assembled by at least this number of tools, default: 2;
—min_seed/-d <float>	Min seed coverage for extension, default: 0;
—temp_dir/-T <string>	Directory storing temporary files, default: ./TransBorrow_tmp;
—threads/-t <int>	Number of threads to launch, default: 1;
—version/-v	Show current version of TransBorrow;
—help/-h	help infomation;

The followings are some important parameter descriptions and suggestions for using TransBorrow to assemble transcripts.

1. According to data types, you need to choose the appropriate parameter “-s”, e.g. for the data SRR7807492 that is paired-end and nonstranded, adjust the parameter to “-s unstranded”.
2. The parameter “-c” indicates the minimum coverage of recovered transcripts, which helps to filter out potentially low-confidence transcripts.
3. The parameter “-l” indicates the minimum length (bp) of recovered transcripts. This parameter helps to filter out short transcripts.
4. The parameter “-d” refers to the minimum seed coverage in the path-extensions procedure of the TransBorrow algorithm. The default value for this parameter is typically set to 0. A higher value of min_seed for extension will result in more stringent filtering, potentially excluding low-coverage regions and leading to fewer but more confident assembled transcripts.

#### Assembling with StringTie, Cufflinks, and Scallop, respectively

##### Assembling with StringTie

To run StringTie, begin by navigating to the StringTie directory and moving the three mapping files in BAM format to the current directory. Then, execute the “stringtie” command to conduct the transcript assembly process, the assembled transcripts will be generated in GTF format (see the following illustration). Moreover, it is essential to adjust the parameters according to the provided data types. The recommended command using StringTie is as follows.

# stringtie -p 8 -o OutputFile.gtf InputFile.bam

In this command, “-p” specifies the number of threads to be used, which is typically set to be equal to or slightly fewer than the number of available CPU cores. The “-o” parameter specifies the name of the output GTF file, and InputFile.bam indicates the name of the input BAM file. The resulting assembly GTF file will be saved in the current folder.

An illustration of running StringTie with its default sets is shown below.

$ cd Stringtie

$ mv/mnt/data0/zhaody/Samtools/SRR7807492_genome.bam.

$ mv/mnt/data0/zhaody/Samtools/ERR3639851_genome.bam.

$ mv/mnt/data0/zhaody/Samtools/SRR10611964_genome.bam.

$ stringtie -p 8 -o SRR7807492_genome_stringtie.gtf SRR7807492_genome.bam

$ stringtie -p 8 -o ERR3639851_genome_stringtie.gtf ERR3639851_genome.bam

$ stringtie -p 8 -o SRR10611964_genome_stringtie.gtf SRR10611964_genome.bam

##### Assembling with Cufflinks

To assemble the transcripts by Cufflinks, navigate to the Cufflinks directory and move the three mapping files in BAM format to the current directory. Subsequently, execute the “cufflinks” command to carry out the transcript assembly, which will generate three GTF files containing the assembled transcripts. It is imperative to adjust the commands accordingly for the provided data types (see the following illustration). The recommended command using Cufflinks is as follows.

# cufflinks -p 8 -o OutputFile.gtf InputFile.bam—library-type fr-firststrand

In this command, the “-p” parameter specifies the number of threads to be used, typically matching or slightly lower than the available CPU cores. The “-o” parameter is followed by the desired name for the output GTF file and the name of the input BAM file. The “—library-type fr-firststrand” parameter indicates that the input RNA-seq data were generated using the first-strand cDNA synthesis method. Conversely, the “—library-type rf-secondstrand” parameter would indicate that the input RNA-seq data was generated using the second-strand cDNA synthesis method. The default assumption for input RNA-seq data is nonstranded.

An illustration of running Cufflinks with its default sets is shown below.

$ cd Cufflinks

$ mv/mnt/data0/zhaody/Stringtie/SRR7807492_genome.bam.

$ mv/mnt/data0/zhaody/Stringtie/ERR3639851_genome.bam.

$ mv/mnt/data0/zhaody/Stringtie/SRR10611964_genome.bam.

$ cufflinks -p 8 -o SRR7807492_genome_cufflinks.gtf SRR7807492_genome.bam

$ cufflinks -p 8 -o ERR3639851_genome_cufflinks.gtf ERR3639851_genome.bam

$ cufflinks -p 8 -o SRR10611964_genome_cufflinks.gtf SRR10611964_genome.bam—library-type fr-firststrand

##### Assembling with Scallop

To assemble the transcripts by Scallop, begin by navigating to the Scallop directory and moving the three mapping files in BAM format to the current directory. Next, execute the “scallop” command to perform transcript assembly, and the assembled transcripts will be generated in GTF format. It is important to note that Scallop requires different parameters to be configured based on the specific data type of the input. Therefore, it is necessary to adjust the commands accordingly for the provided data types. The recommended command using Scallop is as follows.

# scallop -i InputFile.bam -o OutputFile.gtf—library-type first

In this command, the “-i” parameter is followed by the name of the input BAM file, and the “-o” parameter is followed by the desired name for the output GTF file. The “—library-type first” parameter indicates that the input RNA-seq data were generated using the first-strand cDNA synthesis method. Conversely, the “—library-type second” parameter would indicate that the input RNA-seq data were generated using the second-strand cDNA synthesis method. Additionally, the “—library-type unstranded” parameter indicates that the input RNA-seq data are nonstranded. By default, the final assembly GTF file will be saved in the current directory.

An illustration of running Scallop with its default sets is shown below.

$ cd Scallop

$ mv/mnt/data0/zhaody/Cufflinks/SRR7807492_genome.bam.

$ mv/mnt/data0/zhaody/Cufflinks/ERR3639851_genome.bam.

$ mv/mnt/data0/zhaody/Cufflinks/SRR10611964_genome.bam.

$ scallop -i SRR7807492_genome.bam -o SRR7807492_genome_scallop.gtf—library_type unstranded

$ scallop -i ERR3639851_genome.bam -o ERR3639851_genome_scallop.gtf—library_type unstranded

$ scallop -i SRR10611964_genome.bam -o SRR10611964_genome_scallop.gtf—library_type first

#### Merging of assembly files generated from different assemblers

Besides the alignment file, TransBorrow needs to take as input the transcripts assembled by different transcript assembly tools, which should be merged together first. Create a new directory named “Gtf_file” in the home directory to store the merged files. Next, move the nine GTF files to the current directory. Finally, utilize the cat command to merge the three GTF files corresponding to each sample into one file (see the following illustration).

An illustration of merging the assembled transcripts of different assemblers is shown as follows.

$ mkdir Gtf_file

$ cd Gtf_file

$ mv/mnt/data0/zhaody/Stringtie/SRR7807492_genome_stringtie.gtf.

$ mv/mnt/data0/zhaody/Cufflinks/SRR7807492_genome_cufflinks.gtf.

$ mv/mnt/data0/zhaody/Scallop/SRR7807492_genome_scallop.gtf.

$ cat SRR7807492_genome_stringtie.gtf SRR7807492_genome_cufflinks.gtf

SRR7807492_genome_scallop.gtf > combine_SRR7807492.gtf

#### Assembling transcripts for each sample with TransBorrow

To utilize TransBorrow, navigate to the TransBorrow directory and move the mapping BAM files, reference genome FASTA file, and the merged transcripts for each sample to the current directory. Employ the “TransBorrow” command to perform the transcript assembly procedure (see the following illustration for details). It is important to consider that different parameters need to be configured for different types of sample data.

The recommended command using TransBorrow is as follows.

# TransBorrow -r CombinedInputFile.gtf -b InputFile.bam -g ref_genome.fa -s first -o OutputDirctory

In this command, the “-r” parameter is followed by the combined transcripts assembled by different tools in GTF format. The “-b” parameter is followed by the name of the input BAM file. The “-g” parameter is followed by the reference genome in FASTA format. The parameter “-s” indicates the type of sequencing data, whether it is stranded or nonstranded, single-ended or paired-ended. In this case, “first” indicates that the sequencing data were generated using the first-strand cDNA synthesis method. The “-o” parameter is followed by a directory that stores the temporary files and the final assembled GTF file. Please refer to Table 2 for more details.

An illustration of running TransBorrow is shown as follows.

$ cd TransBorrow

$ mv/mnt/data0/zhaody/Gtf_film/combine_SRR7807492.gtf.

$ mv/mnt/data0/zhaody/Gtf_film/combine_ERR3639851.gtf.

$ mv/mnt/data0/zhaody/Gtf_film/combine_SRR10611964.gtf.

$ mv/mnt/data0/zhaody/Scallop/SRR7807492_genome.bam.

$ mv/mnt/data0/zhaody/Scallop/ERR3639851_genome.bam.

$ mv/mnt/data0/zhaody/Scallop/SRR10611964_genome.bam.

$ mv/mnt/data0/zhaody/Hisat2/ref_index_genome.

$ TransBorrow -r combine_SRR7807492.gtf -b SRR7807492_genome.bam -g ref_genome.fa -s unstranded -o ./TransBorrow_results/SRR7807492_TransBorrow.gtf -n 3 -t 8

$ TransBorrow -r combine_ERR3639851.gtf -b ERR3639851_genome.bam -g ref_genome.fa -s single_unstranded -o ./TransBorrow_results/ERR3639851_TransBorrow.gtf -n 3 -t 8

$ TransBorrow -r combine_SRR10611964.gtf -b SRR10611964_genome.bam -g ref_genome.fa -s first -o ./TransBorrow_results/SRR10611964_TransBorrow.gtf -n 3 -t 8

**Figure 5.**
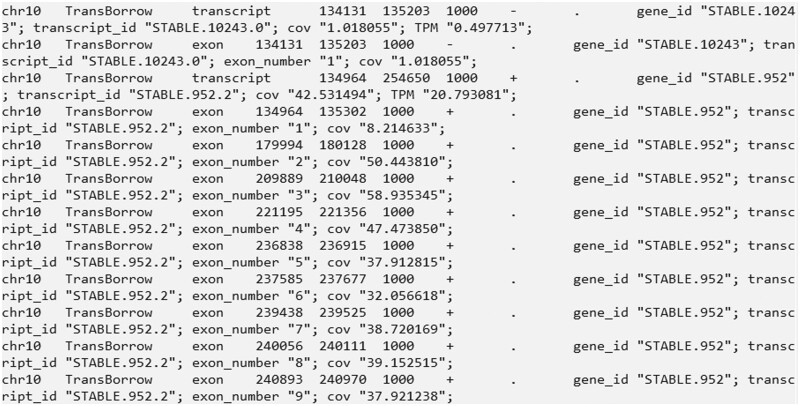
A snapshot of several lines in the SRR7807492_TransBorrow.gtf file that stored the assembled transcripts. The file includes the name of the chromosome, the source of the annotation (TransBorrow), the type of the annotation (“transcript” or “exon”), the start and end positions of the annotation; the score or confidence level of the annotation (usually 1000), the direction of the strand of the annotation (with “+” representing the positive strand, “-” representing the negative strand, and “.” indicating no strand information), gene_id (the index of a gene), transcript_id (the index of a transcript), exon_number (the index of exons), cov (estimated coverage), and transcripts Per Million (TPM).

Finally, the results are saved in the directory “./TransBorrow_results/” (see Fig. 5 for an illustration of the GTF file).

### Transcripts statistics

#### Detecting the correctly assembled transcripts using Gffcompare (optional)

Note that it is often impossible to obtain the expressed transcripts and their expression levels for real data. However, an assembler can be considered more sensitive if it assembles a higher number of transcripts that match known annotations from published gene databases. It can also be viewed as more precise if the known transcripts represent a higher proportion of all transcripts assembled by the assembler. Therefore, to evaluate the performance of an assembler, we used all the transcripts in the annotation library as the ground truth. Gffcompare was used to detect the correctly assembled transcripts, and an assembled transcript is regarded as correctly assembled if and only if its intron chain is exactly matched with an annotation transcript [19].

To evaluate the performance of TransBorrow, navigate to the Gffcompare directory and move the three assembly files in GTF format to the current directory. In addition, download the appropriate reference transcript annotations and unzip the “.gz” file. Subsequently, execute the “gffcompare” command to detect the correctly assembled transcripts using Gffcompare, which will generate six files containing the statistical information.

The recommended command using Gffcompare is as follows.

# gffcompare -G -r ReferenceAnnotation.gtf InputFile.gtf -o OutputFilePrefix

**Figure 6.**
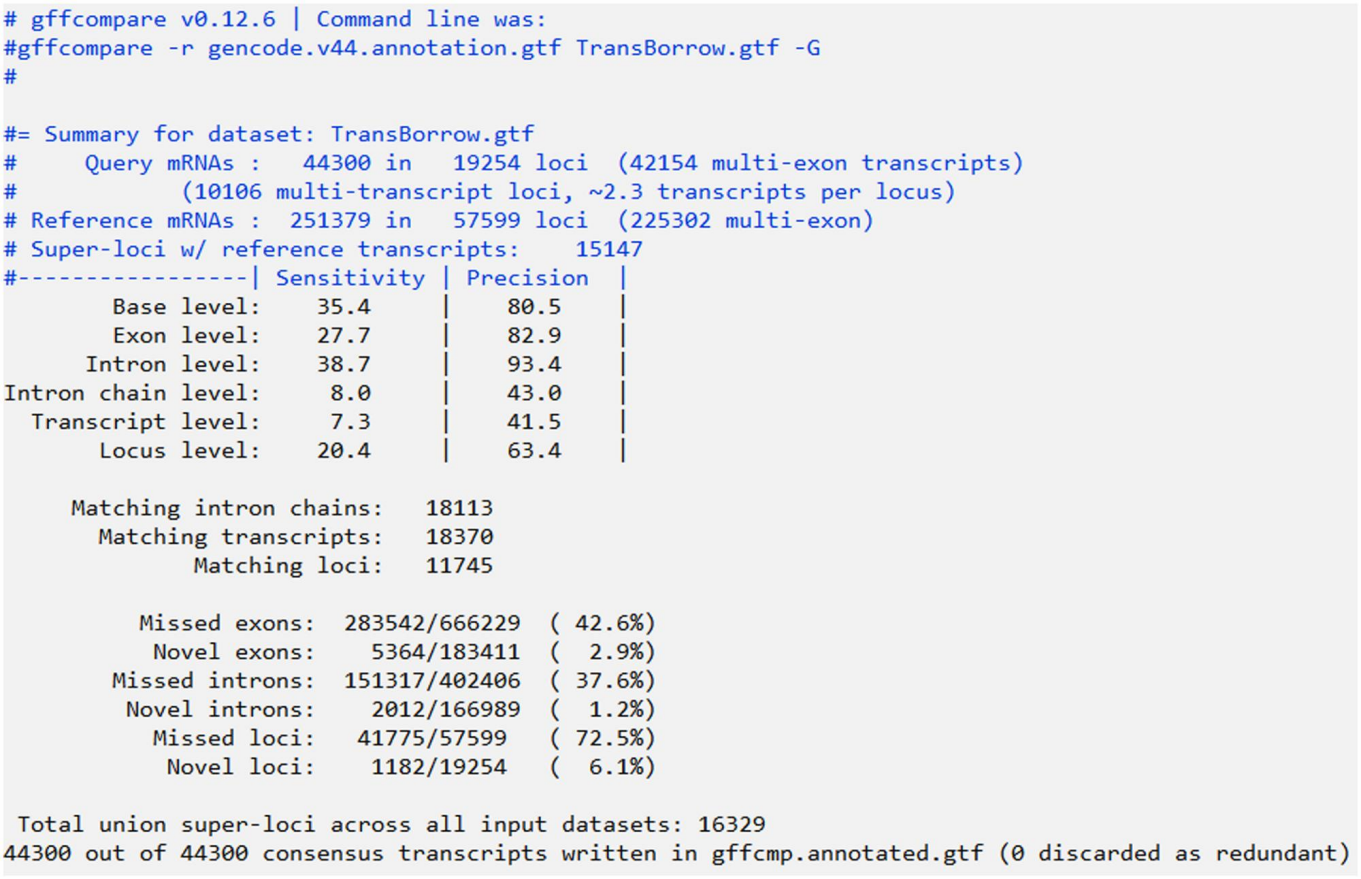
A snapshot of the SRR7807492_TransBorrow.stats file that stored the evaluation results of TransBorrow assembled results for the dataset SRR7807492. According to the output, the number of candidate transcripts was 44,300 and the number of transcripts that correctly match known annotated transcripts was 18,370, with a sensitivity/recall of 20.4% from the locus level and a precision of 41.5% from the transcript level.

In this command, the “-G” parameter specifies to compare all transcripts in the input GTF file, even if they are potentially redundant. The “-r” parameter is followed by the GTF file of the reference transcript annotation, and the InputFile.gtf is the GTF file containing the input transcripts. The “-o” parameter is followed by the prefix of the output files. By default, the final output files generated by Gffcompare include OutputFilePrefix.annotated.gtf, OutputFilePrefix.loci, OutputFilePrefix.stats, OutputFilePrefix.tracking, OutputFilePrefix.gtf.refmap, and OutputFilePrefix.gtf.temp. Among these files, the OutputFilePrefix.stats file contains data summaries and accuracy assessments, including Sensitivity and Precision assessments at six levels: Base level, Exon level, Intron level, Intron chain level, Transcript level, and Locus level (see Fig. 6 for an illustration of gffcompare output).

An illustration of running Gffcompare is shown as follows.

$ cd Gffcompare

$ wget https://ftp.ebi.ac.uk/pub/databases/gencode/Gencode_human/release_44/gencode.v44.annotation.gtf.gz

$ gunzip gencode.v44.annotation.gtf.gz

$ mv/mnt/data0/zhaody/TransBorrow/TransBorrow_results/SRR7807492_TransBorrow.gtf.

$ mv/mnt/data0/zhaody/TransBorrow/TransBorrow_results/ERR3639851_TransBorrow.gtf.

$ mv/mnt/data0/zhaody/TransBorrow/TransBorrow_results/SRR10611964_TransBorrow.gtf.

$ gffcompare -G -r human_annotation.gtf SRR7807492 TransBorrow.gtf -o SRR7807492 TransBorrow

$ gffcompare -G -r human_annotation.gtf ERR3639851_TransBorrow.gtf -o ERR3639851_TransBorrow

$ gffcompare -G -r human_annotation.gtf SRR10611964_TransBorrow.gtf -o SRR10611964_TransBorrow

#### Comparing Transborrow to two ensemble approaches TACO and StringTie-merge

We also tested two ensemble transcriptome assemblers, StringTie-merge and TACO, which were designed to generate a consensus transcriptome from multiple RNA-seq samples. Here, we ran StringTie-merge and TACO by incorporating assemblies generated by different assemblers, including StringTie, Scallop, and Cufflinks (as those are from different samples).

##### Details of running TACO

To merge the transcripts by TACO, navigate to the TACO directory and move the three assembled GTF files generated by different assemblers to the current directory. Then, add the absolute paths of all GTF files to a TXT file as the input file for TACO. Subsequently, execute the “taco_run” command to carry out the transcript merging, which will generate a GTF file containing the merged transcripts. The recommended command using TACO is as follows.

# taco_run gtf_files.txt—filter-min-expr 0.001 -o TACO_OutDirctory -p 8

In this command, the “taco_run” command specifies the input TXT file. The “-p” parameter specifies the number of threads to be used, typically matching or slightly lower than the available CPU cores. The “-o” parameter is followed by the desired name for the output GTF file. The “—filter-min-expr” parameter is used to filter redundant transcripts to retain only those with expression levels above the specified threshold, and it is recommended here that a setting of 0.001 will provide more flexibility in capturing transcripts with low expression levels.

An illustration of running TACO with its default sets is shown below.

$ cd TACO

$ mv/mnt/data0/zhaody/Gtf_file/SRR7807492_genome_stringtie.gtf.

$ mv/mnt/data0/zhaody/Gtf_file/SRR7807492_genome_cufflinks.gtf.

$ mv/mnt/data0/zhaody/Gtf_file/SRR7807492_genome_scallop.gtf.

$ ls -R/mnt/data0/zhaody/TACO/SRR7807492_genome.* > SRR7807492_genome_merge.txt

$ ls -R/mnt/data0/zhaody/TACO/ERR3639851_genome.* > ERR3639851_genome_merge.txt

$ ls -R/mnt/data0/zhaody/TACO/SRR10611964_genome.* > SRR10611964_genome_merge.txt

$ taco_run SRR7807492_genome_merge.txt—filter-min-expr 0.001 -o ./SRR7807492_genome_merge.gtf

$ taco_run ERR3639851_genome_merge.txt—filter-min-expr 0.001 -o ./ERR3639851_genome_merge.gtf $ taco_run SRR10611964_genome_merge.txt—filter-min-expr 0.001 -o ./SRR10611964_genome_merge.gtf

##### Details of running StringTie-merge

To merge the transcripts by StringTie-merge, navigate to the StringTie directory and move the three assemblies’ GTF file generated by different assemblers to the current directory. Subsequently, execute the “stringtie-merge” command to carry out the transcript merging, which will generate a GTF file containing the merged transcripts. The recommended command using StringTie—merge is as follows.

# stringtie—merge stringtie.gtf scallop.gtf cufflinks.gtf -T 0.001 -F 0.001 -o StringTie_merge.gtf

In this command, the “—merge” parameter is followed by three input GTf files. The “-o” parameter is followed by the desired name for the output GTF file. The parameter “-T” and “-F” indicates the minimum TPM and FPKM values of transcripts to be included in the merged results, and a setting of 0.001 is recommended here to allow for more transcripts with lower expression to be merged.

An illustration of running StringTie-merge with its default sets is shown below.

$ cd Stringtie

$ mv/mnt/data0/zhaody/Gtf_file/SRR7807492_genome_stringtie.gtf.

$ mv/mnt/data0/zhaody/Gtf_file/SRR7807492_genome_cufflinks.gtf.

$ mv/mnt/data0/zhaody/Gtf_file/SRR7807492_genome_scallop.gtf.

$ stringtie—merge SRR7807492_genome_stringtie.gtf SRR7807492_genome_cufflinks.gtf SRR7807492_genome_scallop.gtf -T 0.001 -F 0.001 -o SRR7807492_StringTie_merge.gtf

$ stringtie—merge ERR3639851_genome_stringtie.gtf ERR3639851_genome_cufflinks.gtf ERR3639851_genome_scallop.gtf -T 0.001 -F 0.001 -o ERR3639851_StringTie_merge.gtf

$ stringtie—merge SRR10611964_genome_stringtie.gtf SRR10611964_genome_cufflinks.gtf SRR10611964_genome_scallop.gtf -T 0.001 -F 0.001 -o SRR10611964_StringTie_merge.gtf

## Results

In this protocol, we used three RNA-seq data to evaluate the performance of the assembly tools. Two of the latest mapping software, Hisat2 and Star [17], were employed to align the raw RNA-seq data to the reference genome. We compared TransBorrow with StringTie, Cufflinks, and Scallop. Additionally, we included the comparison of two merge-based assemblers, namely StringTie-merge and TACO [19, 20].

To evaluate the performance of an assembler, we used the following metrics: the number of correctly assembled transcripts, precision, recall, and *F*-score, where the precision is defined as the percentage of the correctly assembled transcripts out of the candidates and recall is defined as the fraction of the correctly recovered transcripts in the ground truth, and *F*-score is a harmonic mean of recall and precision (calculated as 2*precision*recall/(precision + recall)) [21, 22].

### TransBorrow performs the best in assembling both single-end and paired-end RNA-seq data

**Figure 7.**
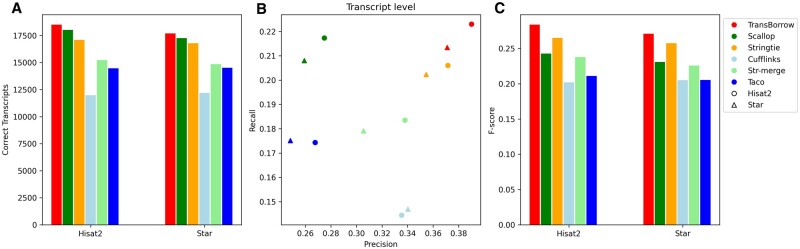
Performance comparisons of the assemblers on the SRR7807492 dataset (paired-end and nonstranded). (A) The number of correctly assembled transcripts by the assemblers. (B) Assembly accuracy of the assemblers in terms of precision and recall. (C) F-scores of the assemblers.

**Figure 8.**
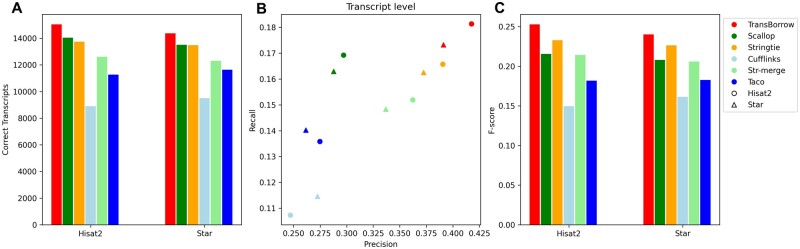
Performance comparisons of the assemblers on the ERR3639851data (single-end and nonstranded). (A) The number of correctly assembled transcripts by the assemblers. (B) Assembly accuracy of the assemblers in terms of precision and recall. (C) F-scores of the assemblers.

First, we compared TransBorrow to the alternatives using the data sets with different sequencing library layouts (i.e. single- or paired-end reads), and the data SRR7807492 (paired-end) and ERR3639851 (single-end) were selected as the test data. After comparison, TransBorrow exhibited superior performance in terms of the four aforementioned metrics when compared to other assemblers (see Figs 7 and 8 for details).

In detail, for the number of correctly assembled transcripts of the paired-end RNA-seq data SRR7807492 under Hisat2 and Star mappings, TransBorrow correctly detected 8.30% and 5.49% more expressed transcripts compared to StringTie, 2.66% and 2.61% more than Scallop, 54.47% and 45.23% more than Cufflinks, 21.57% and 19.17% more than StringTie-merge, and 27.95% and 21.87% more than Taco. Similarly, for the number of correctly assembled transcripts of the single-end RNA-seq data ERR3639851 under the two aligners, TransBorrow correctly identified 9.42% and 6.53% more expressed transcripts than StringTie, 7.13% and 6.31% more than Scallop, 68.94% and 51.13% more than Cufflinks, 13.34% and 16.75% more than StringTie-merge, and 33.48% and 23.47% more than Taco. Meanwhile, in terms of precision, TransBorrow also exhibited significant improvement against the others. Therefore, the *F*-score of TransBorrow reached the highest as well.

These results demonstrated that TransBorrow performs better than the other assemblers in assembling both single-end and paired-end RNA-seq data.

### TransBorrow performs the best in assembling both strand-specific and nonstranded RNA-seq data

**Figure 9.**
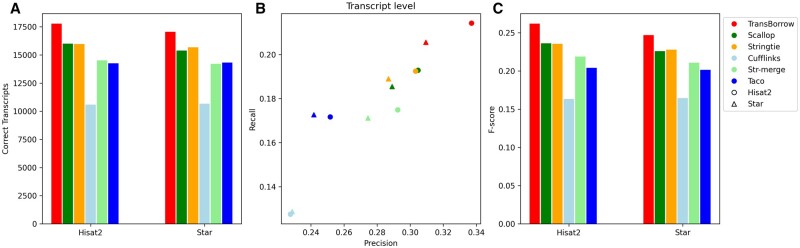
Performance comparisons of the assemblers on the SRR10611964 data (paired-end and strandspecific). (A) The number of correctly assembled transcripts by the assemblers. (B) Assembly accuracy of the assemblers in terms of precision and recall. (C) F-scores of the assemblers.

In the RNA-seq experiment, two kinds of sequencing protocols were generally employed, which were stranded-specific and nonstranded RNA-seq protocols. To assess the performance of the assemblers under different sequencing protocols, we selected two data sets, SRR7807492 (nonstranded) and SRR10611964 (strand specific). We also compared TransBorrow to other assemblers in terms of the four aforementioned metrics. Once again, TransBorrow exhibited superior performance when compared to other assemblers (see Figs. 7 and 9 for details).

In detail, for the correctly assembled transcripts on the nonstranded RNA-seq data SRR7807492 under Hisat2 and Star mappings, TransBorrow demonstrated significant improvement as mentioned above. For the correctly assembled transcripts on the strand-specific RNA-seq data SRR10611964 under the two aligners, TransBorrow detected 11.53% and 8.80% more expressed transcripts than StringTie, 11.08% and 10.83% more than Scallop, 68.04% and 59.81% more than Cufflinks, 22.55% and 20.06% more than StringTie-merge, and 24.78% and 19.06% more than Taco. And, for the precision and *F*-score, TransBorrow also reached the highest. These results provide further evidence that TransBorrow outperformed the other compared assemblers in assembling both strand-specific and nonstranded RNA-seq data.

### TransBorrow consistently performs the best by using different alignment tools

As mentioned, various RNA-seq alignment tools were available for the researchers, and Hisat2 and Star are the two most widely used mappers. In order to evaluate the performance of the assemblers under different alignment tools, we used the two different aligners to generate the mapping results of the RNA-seq data. Then we compared TransBorrow to the others using the three data sets SRR7807492, ERR3639851, and SRR10611964, under both Hisat2 and Star mappings. As compared above, TransBorrow consistently demonstrated the best performance in terms of all the aforementioned accuracy metrics regardless of the alignment tools (see Figs. 7–9 for details).

## Discussion

Developing accurate and efficient transcript assembly software is crucial for effectively processing large-scale RNA sequencing data. It enables further exploration of the transcriptome and investigations into complex human diseases, such as cancers, which are often associated with aberrant splicing events and expression levels [23, 24]. In this protocol, we report TransBorrow, a genome-guided transcriptome assembler specifically designed to assemble RNA sequencing reads into full-length transcripts and estimate expression levels. We show the detailed running process of the tool and compared its performance with those of the same kind. After comparison, the results demonstrated that TransBorrow consistently performs better than all the other compared assembly software in terms of three aspects: assembling single-end or paired-end sequencing data, assembling strand-specific or nonstranded RNA-seq data, and assembling based on different alignment tools.

TransBorrow incorporates several advantages that contribute to its superior performance. First, it effectively borrows the assembly results of other software to guide its own assembly process, which combines the strengths of multiple tools. Second, TransBorrow integrates the assemblies of other assembles to build a novel graph model, namely color graph, which facilitates efficient extraction of reliable subpaths. Third, TransBorrow constructs the weighted line graph based on the splice graph and the extracted reliable subpaths. Fourth, TransBorrow introduces a new strategy for identifying transcript-representing paths from the weighted line graphs.

Although we have seen great advantages taken by TransBorrow, the current version of TransBorrow does have several limitations, such as the inability to assemble long reads, incompatible with *de novo* assemblies, and the relatively complex installation process. In the future, we will definitely tackle these problems.

In conclusion, TransBorrow stands out among existing software by accurately assembling complex raw sequencing data into expressed transcripts. By utilizing TransBorrow, researchers can effectively harness the power of RNA-seq for advanced transcriptome analysis.

## Data and code availability statement

All the data that are presented are included in the article materials and further inquiries can be directed to the corresponding author.
